# Fire-Driven Land Cover Change and Zoonotic Disease Risk in African Landscapes

**DOI:** 10.1007/s10393-025-01743-9

**Published:** 2025-07-25

**Authors:** Ore Koren

**Affiliations:** Department of Political Science, Ostrom Workshop for Political Analysis, and Tobias Center for Innovation in International Development, Indiana University Bloomington, Bloomington, IN USA

**Keywords:** infectious disease, zoonosis, fires, deforestation, geolocated data

## Abstract

Land use and land cover (LULC) change—especially habitat disruption—has long been linked to zoonotic disease emergence, yet direct empirical evidence remains limited. Using new spatially disaggregated data, this study offers the clearest evidence to date that fire-linked vegetation loss significantly increases zoonotic risk in forest landscapes, but not in agricultural or other-use areas. Monthly vegetation anomalies, captured via NDVI deviations, are tracked across landscape types. A quasi-experimental mediation design estimates the indirect effect of fire on outbreaks through vegetation loss. Results show that in forests, fire-driven vegetation decline is associated with increased outbreak rates, while no significant effects appear in agricultural or other zones. Fires—including slash-and-burn practices and uncontrolled natural fires—hence play a key role in shaping zoonotic risk. The results underscore the need for integrated fire management and land-use strategies to reduce spillover potential and align public health with climate and conservation goals.

**Supplementary Information:**

The online version contains supplementary material available at 10.1007/s10393-025-01743-9.

## Introduction

Land use and land cover (LULC) changes are widely suspected to influence the risk of zoonotic disease outbreaks. Researchers have long hypothesized that deforestation and agricultural expansion can increase human exposure to previously isolated pathogens, especially in forest edge zones where wildlife habitats are disturbed (Gottdenker et al. 2014; Allen et al. 2017). Yet empirical tests of this hypothesis remain limited, constrained by coarse or sparse disease datasets or reliant on indirect proxies such as species richness (Randolph and Dobson 2012; Jones et al. 2008). Does zoonotic risk rise mainly due to habitat destruction that forces wildlife into closer proximity with humans (Vinson et al. 2022)? Or is the risk driven by agricultural expansion that supports vector populations, such as rodents and birds (Gottdenker et al. 2014; Hjelle and Glass 2000)? Social, political, and environmental contexts likely shape these outcomes (Koren and Chaves 2025). Without clarity on these pathways, interventions risk being misdirected or counterproductive (Levins 1995). Understanding why and where LULC changes translate into outbreak risk is thus essential for effective and equitable policy.

Historical cases provide suggestive evidence of potential links between LULC change and zoonotic emergence. Hantavirus outbreaks, for example, have been associated with agricultural and forest work, where exposure is thought to occur through inhalation of aerosolized particles contaminated by rodent excreta (Hjelle and Glass 2000; Nichol et al. 1993). A meta-analysis suggests that both agricultural and forestry workers face statistically elevated risks compared to other populations, likely due to their proximity to rodent habitats and disturbance of nesting areas (Riccò et al. 2021). Other pathogens, such as HIV and Ebola, are similarly suspected to have crossed into humans through increased contact with wildlife in forested environments. HIV strains closely resemble the Simian Immunodeficiency Virus (SIV), with some scholars arguing that spillover may have occurred via bushmeat hunting in Central Africa and spread via exposure to non-sanitized healthcare instruments (Pepin 2011; Sharp and Hahn 2010). In the case of Ebola, while the exact origin remains uncertain, transmission from fruit bats or other wildlife during forest exposure has been proposed (Leroy et al. 2005; Judson and Munster 2023). Agriculture-related land use is also implicated in some accounts: rabies transmission from vampire bats to humans and livestock in South America has been linked to farming near forest edges (Belotto et al. 2005), and the expansion of rice and pig farming is believed to have facilitated the spread of Japanese encephalitis via mosquito vectors (Van den Hurk et al. 2009; Koren et al. 2024). While these examples cannot confirm causal pathways, they highlight plausible routes through which LULC changes might facilitate zoonotic spillover.

Among the many pathways linking land use to zoonotic emergence, fire – whether from natural causes or slash-and-burn practices—plays a potentially critical but under-analyzed role. Fires can rapidly alter vegetation structure, reduce habitat quality, and drive wildlife into closer proximity with human populations (Scasta 2015; Ecke et al. 2019). These effects are expected to be particularly pronounced in agricultural and forested landscapes. In agricultural zones, fires that clear land and destroy food sources, encouraging species such as rodents and wild boars—common disease vectors—to enter farming areas in search of resources and to escape the disruptions from the fire. In forested regions, habitat disruption from fire can displace bats and birds known to carry viruses like Ebola, potentially pushing them into closer contact with human populations (Jones et al. 2013). Areas situated along forest edges, where human activity overlaps with critical wildlife habitats, may be especially vulnerable to such dynamics. Yet, despite its relevance, fire has rarely been examined directly as a systematic driver of zoonotic outbreaks across diverse ecological contexts (for some studies that touched on this topic see: Ecke et al. 2019; Ruiz et al. 2019; Scasta 2015).

Using new data, this study provides the clearest empirical evidence to date of how fire-driven land use and land cover (LULC) changes influence zoonotic disease. The analysis draws on the Geolocated Zoonotic Outbreak Dataset (GZOD), a recently published event-based dataset that records the location and timing of zoonotic outbreaks across Africa (Koren and Bukari 2024). By linking these outbreak events with satellite-derived measures of vegetation loss and fire activity, the study evaluates how fire-induced LULC change mediates disease risk across agricultural, forest, and transitional zones. In doing so, it provides a pertinent spatially explicit test of a long-standing hypothesis: that environmental disturbance—particularly via fire—can heighten zoonotic spillover risk by reshaping habitats and intensifying contact between humans and wildlife.

## Methods

### Data

This study uses a monthly panel dataset of 0.5-degree (~ 55 km) grid cells across Africa from January 2003 to December 2018, harmonized using the AfroGrid framework (Schon & Koren 2022). The unit of analysis strikes a balance between spatial precision and data reliability, given the limitations of outbreak reporting in rural regions (Weidmann 2015).

The dependent variable is based on the Geolocated Zoonotic Outbreak Dataset (GZOD), which records N = 512 WHO- or ISID-confirmed outbreaks across Africa involving pathogens with strong animal reservoirs, such as Ebola, Marburg, plague, rabies, anthrax, and influenzas (Koren & Bukari 2024). These outbreaks are aggregated at a monthly 0.5° grid resolution to align with AfroGrid. Agricultural zones were identified based on major crop production (e.g., cereals, sugar, bananas) sourced from the Spatial Production Allocation Model (Yu et al. 2020), while forest zones were defined using > 50% forest cover per grid cell based on high-resolution PALSAR data (JAXA) (~ 19.5% of all grid cell months in the sample) (Shimada et al. 2014). Both layers are fixed across time using a carry-forward approach. A map of spatial coverage appears in Fig. S1, SI File.

Fire and vegetation change are modeled as standardized anomalies. NDVI anomalies capture short-term vegetation disruptions relative to long-term local means (Busetto and Ranghetti 2016), while fire anomalies are derived from MODIS fire detections (Justice et al. 2002), also standardized per grid cell and month. Standardizing NDVI and fire measures enhances comparability across ecosystems, isolates meaningful short-term disturbances relative to local norms, and improves temporal specificity for model estimation. This approach is better suited to identifying causal pathways, as it emphasizes dynamic shocks—rather than static conditions—that are most relevant for understanding zoonotic spillover processes. Additional covariates include log-transformed nighttime light (as a proxy for development and state presence) from the Defense Meteorological Satellite Program’s Operational Linescan System (DMSP-OLS) system, with corrections applied via the more sensitive (especially in rural areas) Visible Infrared Imaging Radiometer Suite (VIIRS) method (Li et al. 2020); and population density from the WorldPop dataset, which is advantageous for analyzing population dynamics in remote and rural areas (Tatem 2017). Detailed discussion on the sample and the construction of all variables are provided in the SI File.

### Mediation Analysis

To assess whether changes in land-use and land-cover (LULC) mediate the relationship between fire activity and zoonotic disease outbreaks, I employed a generalized causal mediation framework (Imai et al. 2010), which builds upon earlier work in mediation analysis (Baron and Kenny 1986). This approach enables a formal decomposition of total effects into indirect (mediated) and direct components, while allowing for potential endogeneity of the mediator. Fire anomalies are conceptualized as exogenous shocks to zoonotic disease (i.e., unaffected by outbreaks) that trigger vegetation loss and land-cover changes, which may in turn influence the likelihood of zoonotic spillover. The analysis was implemented using two regression models estimated separately for three LULC categories: (1) agricultural grid cell months, (2) forest grid cell months, and (3) all other grid cell months, where the mechanism is theoretically absent. The first model estimates the effect of fire anomalies on vegetation cover anomalies (mediator); the second estimates the effect of both fire and vegetation anomalies on the likelihood of confirmed zoonotic outbreaks (outcome). The equations are specified as:

1 $$ n_{it} = \alpha_{1} + \beta_{1w} w_{it} + \beta_{1y} y_{it - 1} + \beta_{1p} p_{it} + \beta_{1l} l_{it} + m_{{1_{t} }} + \varepsilon_{1i} $$

2 $$ y_{it} = \alpha_{2} + \beta_{2n} n_{it} + \beta_{2w} w_{it} + \beta_{2y} y_{it - 1} + \beta_{2p} p_{it} + \beta_{2l} l_{it} + m_{{2_{t} }} + \varepsilon_{2i} $$

In Eq. 1, the dependent variable $${n}_{it}$$ represents standardized NDVI anomalies (the mediator), and the key predictor $${w}_{it}$$ measures standardized fire anomalies. $${p}_{it}$$ and $${l}_{it}$$ are controls for population densities and nighttime light emissions, respectively, and $${y}_{it-1}$$ is the lagged disease rates (in the environmentally adjusted model, controls for water deficits/drought and precipitation and temperature anomalies are added). Equation 2 regresses the zoonotic outbreak indicator $${y}_{it}$$  on both the mediator$${n}_{it}$$ and the fire anomaly treatment $$ w_{{it}}  $$ and the controls. Monthly fixed effects $$ m_{t}$$are included to account for seasonal variation; $${\alpha }$$ are the intercepts and $${\epsilon }_{i}$$ are standard errors clustered by grid cell (or country in the country-heterogeneity adjusted model) to account for heterogeneities within the cross-sectional unit (0.5 grid or country) over time, e.g., due to similar repeated values measured.

Under this identification strategy, the assumption is that fire events influence zoonotic disease risks primarily by inducing changes in vegetation and land cover, especially within agricultural and forested landscapes. To detect mediation, I test the significance of the indirect path from fire to vegetation (${\beta }_{1w}\ne 0$) and from vegetation to outbreaks (${\beta }_{2n}\ne 0$), while evaluating the reduction in the direct path (${\beta }_{2w}=0$) once the mediator is included as an explanatory variable in Eq. 2. Inference is based on 1,000 simulations with quasi-Bayesian approximation and standard errors clustered at the grid cell (or country for robustness), estimated using a non-parametric procedure that provides bias-corrected confidence intervals and improved handling of measurement error relative to traditional methods (Imai et al. 2010).

## Results

**Figure 1:**
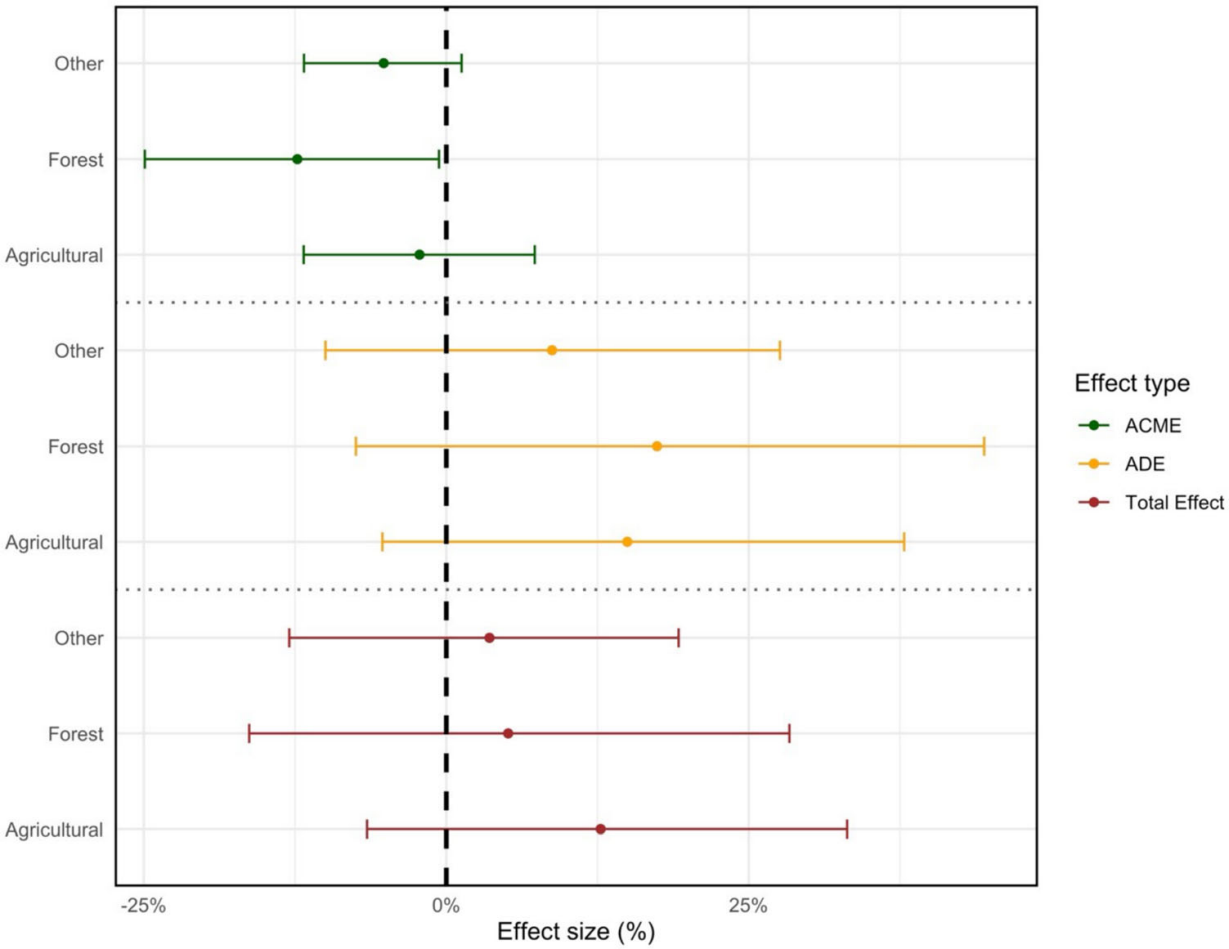
Average Causal Mediation Effect as percent change from zoonotic outbreak mean (ACME, top), Average Direct Effect (ADE, center), and Total Effects (bottom) for vegetation anomalies in agricultural, forest, and other (non-agricultural/non-forest) areas in Africa, Jan. 2003–Dec. 2018. Each figure was estimated based on 1000 simulations with grid-cell clustered standard errors. N = 1,114,130 (other), N = 363,494 (forest), N = 556,372 (agricultural). P-values (two-tail tests) are *p* = 0.100 (ACME, other), *p* = 0.042 (ACME, forest), *p* = 0.506 (ACME, agricultural), p = 0.356 (ADE, other), *p* = 0.296 (ADE, forest), *p* = 0.162 (ADE, agricultural), *p* = 0.664 (TE, other), *p* = 0.744 (TE, forest), *p* = 0.520 (TE, agricultural).

Figure 1 summarizes the results of a robust mediation analysis approach (Imai et al. 2010) to test whether fire-induced vegetation loss drives zoonotic outbreaks. Each panel groups three land types (agriculture, forest, other uses): the top panel reports the average causal mediation effects (ACME) of vegetation anomalies via fires, the middle panel shows the average direct effects (ADE) of fire anomalies operating through other channels, and the bottom panel displays total effects (ACME + ADE). The plot includes 95% confidence (based on grid clustering) intervals to assess statistical significance (Complete estimates from these models are provided in Table S2, SI File).

The results show a clear contrast across land types. In forest zones, the ACME is statistically significant—its 95% confidence interval does not include zero—indicating that vegetation loss caused by fires significantly increases zoonotic outbreak risk. This supports the hypothesis that fire-driven habitat degradation displaces wildlife (e.g., bats, rodents, birds), increasing human–wildlife contact at forest edges. In contrast, the ACME for agricultural zones is not statistically significant, suggesting that fire-driven crop loss does not affect zoonotic risk. Coefficient equality tests indicate that the vegetation mediation effect in forest areas is significantly different from that in agricultural areas (*p* < 0.01, two-tail test, assuming a widely used significance threshold of *p* < 0.05), while the difference between forest and other zones falls short of statistical significance (*p* = 0.19), suggesting that the effect of fires in forest areas may be distinct, but not definitively so.

**Figure 2:**
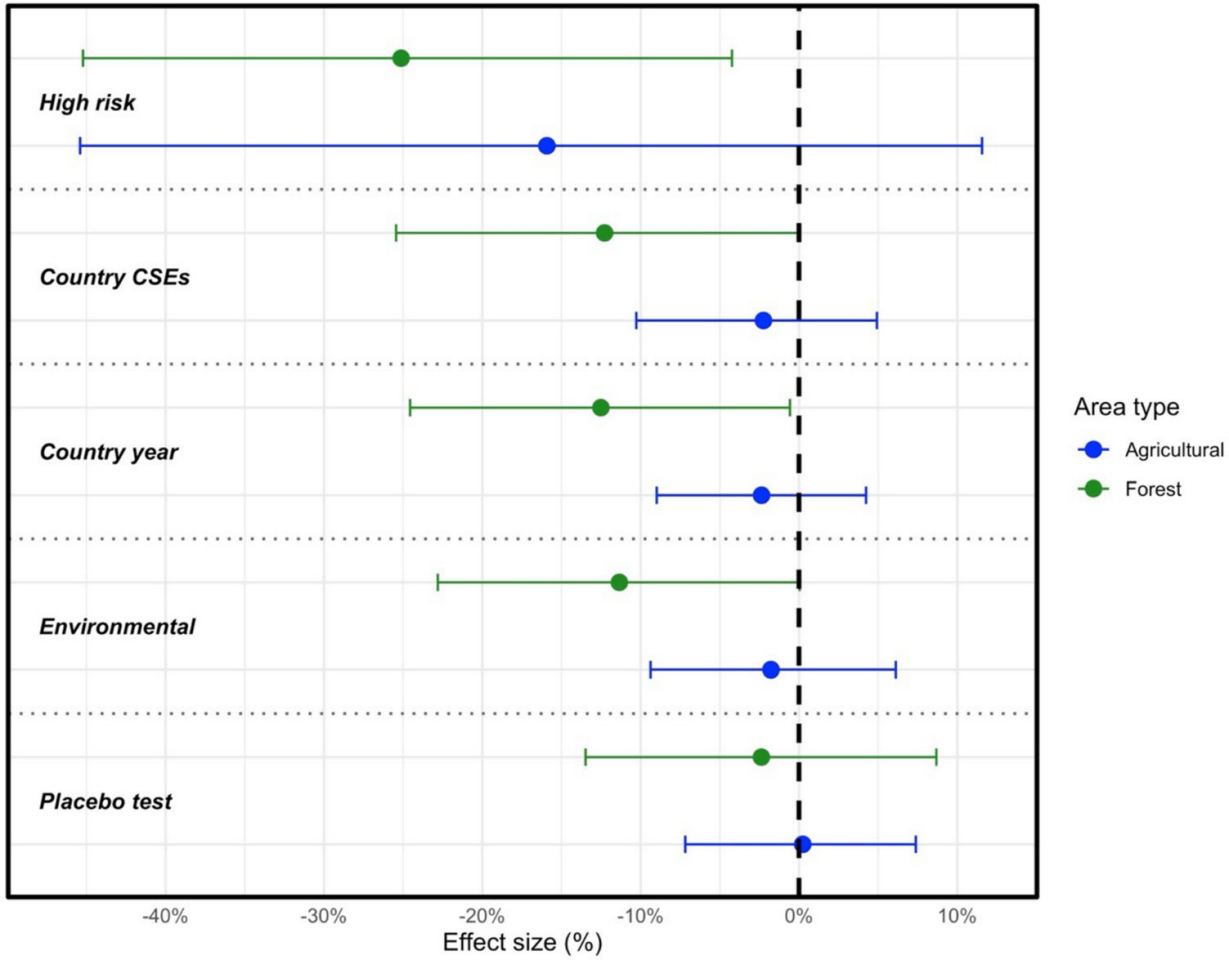
Sensitivity analyses for zoonotic disease ACME for vegetation changes in agricultural and forest zones. (Top) High risk scenario (forest: N = 277,940, *p* = 0.016; agricultural: N = 378,212, *p* = 0.268); (second from top) Country heterogeneities (forest: N = 363,494, *p* = 0.056; agricultural: N = 556,372, *p* = 0.584); (third from top) Country-year adjusted (forest: N = 363,494, *p* = 0.026; agricultural: N = 556,372, *p* = 0.518); (second from bottom) Environmentally-adjusted (forest: N = 394,052, *p* = 0.052; agricultural: N = 478,468, *p* = 0.674); (bottom) Placebo test (forest: N = 277,940, *p* = 0.678; agricultural: N = 85,554, *p* = 0.914).

Sensitivity analyses and placebo tests further support these findings (see full discussion in the SI File). For ease of assessment, the ACME for agricultural and forest areas for each test are reported in Fig. 2. First, to rule out alternative pathways—such as increased human mobility or economic disruption due to fires—a series of placebo tests were first conducted using cell-months from areas with either very low population density (< 1000 people) or large urban centers (population ≥ 50,000). These contexts are unlikely to support the hypothesized mechanism of fire-driven habitat disruption leading to increased human—vector interaction, either due to insufficient population exposure and the density needed for disease spread (rural zones) or physical distance from habitat edges (urban zones). The ACME estimates for agricultural and forest zones drop to near zero in both sparse and urbanized settings, where the mediation effect is not expected to operate, bolstering the interpretation that the effects observed in Fig. 1 reflect true vegetation-mediated LULC pathways, rather than confounding influences related to infrastructure damage or population (displacement Table S3 and Fig. S3).

Additional sensitivity analyses confirm the robustness of the mediation results across alternative specifications (see Methods for a more detailed discussion). First, restricting the sample to moderately populated zones (1,000–50,000 people) excluded from the placebo tests reveals stronger mediation effects, consistent with greater zoonotic risk at the interface of land-use change and human presence (Fig. S4 and Table S4). Second, to rule out national-level confounders such as political and economic features, models were re-estimated using country and year fixed effects; the core results remained stable (Fig. S5 and Table S5). Third, re-estimating the models with standard errors clustered at the country level—rather than the grid-cell level—to account for broader spatial or policy-related heterogeneity produced consistent results, indicating that the observed effects are not biased by national-level correlations (Fig. S6 and Table S6). Finally, accounting for environmental factors—drought, water deficits, and anomalies in rainfall and temperature (Harris et al. 2020)—does not alter the observed effect of fires on zoonotic outbreaks via their influence on vegetation anomalies (Fig. S7 and Table S7).

## Discussion

These findings are particularly salient amid evolving patterns of human–environment interaction, not only in sub-Saharan Africa, but across the globe (Jones et al. 2013). In recent decades, large swaths of the Sahel—the region south of the Sahara Desert that stretches across the entire continent—have experienced vegetation greening, largely due to increased rainfall and flooding. In contrast, areas undergoing sustained browning have done so primarily as a result of fire, much of it manmade, driven by slash-and-burn agriculture or intentional brush clearing for land conversion (Zeng et al. 2023). While some of this activity reflects small-scale land management, the expansion of agricultural frontiers through deliberate forest burning can be ecologically disruptive and has long been hypothesized to induce to elevated disease risks (Walsh et al. 1993). As deforestation and fire activity intensify from human settlement encroachment or agricultural expansion there is mounting concern that such land-use changes may fragment habitats and displace vector species. These shifts increase the likelihood of human–wildlife contact at forest margins, creating new ecological interfaces for zoonotic spillover.

Interventions intended on addressing zoonotic risks linked to land-use change requires locally informed governance that supports sustainable agriculture and fire management. Strategies such as regulating slash-and-burn practices and promoting agroforestry can reduce both ecological degradation and spillover risk. Yet one-size-fits-all interventions may backfire. For example, unchecked forest regrowth can increase exposure to reservoir species (Koren & Chaves 2025; Levins 2010). Disease emergence is shaped not only by ecological disruption but also by structural inequalities that drive unsustainable land use. Conservation efforts that marginalize local communities may further increase risk by fueling displacement and conflict. A structural One Health approach—aligned with initiatives like REDD + and the Kunming-Montreal Global Biodiversity Framework—can help integrate health, equity, and biodiversity goals in land-use planning (Daszak et al. 2020).

The results also have implications for our understanding of how increasing aridity and climate variability intersect with disease-related ecological processes. Climate shifts can disrupt forest ecosystems not only by drying vegetation and increasing flammability, but also by altering the timing and intensity of fire seasons, conditions that, in turn, affect the composition, abundance, and behavior of vector species (Wilcox & Colwell 2005; Ferreira et al. 2020). As fire regimes potentially become less predictable and more severe under warming trends, the ecological disruptions they cause may amplify the risk of zoonotic spillover by accelerating habitat loss and intensifying human-wildlife contact (Jones et al. 2013). These dynamics suggest that climate adaptation efforts—particularly those focused on fire management and land-use planning—should integrate disease risk as a core component of environmental and public health resilience strategies. In this, the findings complement recent work highlighting how climate and land-use change accelerate cross-species viral transmission (Carlson et al. 2022), reinforcing the need for integrated policy approaches to nature and health.

Due to data limitations, this analysis cannot disaggregate fires by ignition source. However, different fire types may have distinct epidemiological impacts. Human-induced fires—such as slash-and-burn agriculture, brush clearing for settlement, or hunting—often coincide with infrastructure expansion, wildlife displacement, and habitat fragmentation (Iglesias et al. 2022; Zeng et al. 2023). These dynamics might be especially relevant at forest edges, where the observed significant mediation effect of vegetation loss on zoonotic outbreaks may reflect increased human-wildlife contact. In contrast, natural fires driven by drought or heatwaves may be broader in scale but less ecologically disruptive (Hantson et al. 2022). Such differences likely affect not only the spatial extent and intensity of vegetation loss but also fire frequency and habitat recovery dynamics (Driscoll et al. 2021). The lack of a mediation effect in agricultural zones and the stronger results in moderately populated areas reinforce the idea that fire type and context matter. While ignition source data are unavailable here, future research could disaggregate fires by cause and intensity to better assess variation in mediation pathways.

**Figure 3:**
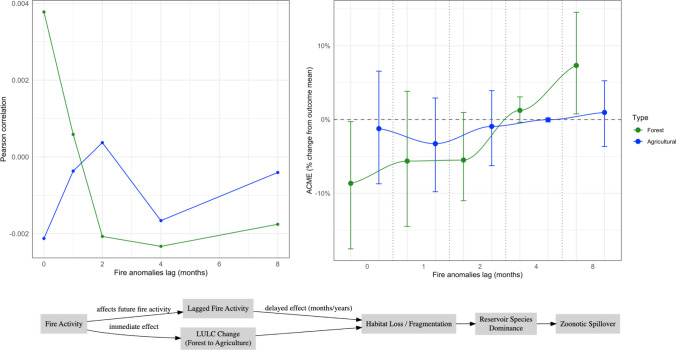
(Top) Pearson correlations between fire anomalies and zoonotic disease outbreak rates (left), and the ACME of contemporaneous vegetation health anomalies (right) based on extended fire anomaly lags in agricultural (blue) and forest (green) zones (Bottom) Hypothesized immediate and lagged dynamics.

Additionally, while the present analysis identifies significant associations between fire activity, land-use/land-cover (LULC) change, and zoonotic disease outbreaks, it does not explicitly account for potential time lags between these processes. Repeated fires may fragment habitats and gradually reduce biodiversity, creating conditions conducive to spillover events months or even years later. To consider these dynamics, Fig. 3 presents correlations between fire anomalies and zoonotic disease rates (top-left) and mediated effect through contemporaneous vegetation health anomaly values (top-right) in agricultural and forest areas at doubling time lags (0, 1, 2, 4, and 8 months), accompanied by a conceptual summary of both immediate and lagged fire-LULC-zoonosis pathways. Zoonosis in forest areas appears to be more influenced by immediate effects, as evidenced by the sharp decline in Pearson correlation values and the cessation of the mediated effect between contemporaneous data and two-month lags. At the same time, Fig. 3 suggests that zoonosis in agricultural areas might be more delayed. Potentially, once a landscape stabilizes into diverse, small- to medium-scale agricultural use, spillover risk plateaus and may be more ecologically resilient to fire-driven changes, though delayed effects during post-fire recovery can create conditions favorable to spillover (Perfecto et al. 2023; Salemi 2021).

Another potential confounder is spatial dependence. Although the analysis uses spatially granular outbreak and vegetation data, such dependencies may influence observed patterns of brush clearing and disease outbreaks. Vegetation loss and zoonotic events are unlikely to be randomly distributed; rather, they may exhibit disease clustering or spillover, where fire-induced degradation or disease risk in one cell increases susceptibility in neighboring areas through ecological connectivity or human mobility (Jones et al. 2013; Koren and Chaves 2025). In mediation models like those used here, such spatial clustering can complicate inference due to endogenous fire and land-use dynamics across space, which require more specific theorization and assessment. While this limitation is acknowledged, simple spatial diagnostics and spatial lag models can help assess robustness under spatial dependence and highlight geospatial processes for future study.

For example, Moran’s I test estimates for temporally collapsed grid level outbreak data suggest no spatial dependence: 0.033 for agricultural areas (mean = 0.709 outbreaks), 0.065 for forest areas (mean = 0.365), and 0.064 for other zones (mean = 0.054). Although none cross the *p* < 0.05 threshold, results for non-agricultural, non-forest zones approach significance (*p* = 0.054), suggesting possible spatial links in outbreak rates in these areas. A more detailed spatial regression mediation model (see Fig. S8 and Table S8) replaces contemporaneous terms with spatial lags for fire and vegetation, and adds a spatial control for zoonotic outbreaks (all created using a k = 4 nearest neighbors algorithm). The results align with the main findings: the ACME of vegetation loss in forest areas remains significant, suggesting that fires push vector species into adjacent forest zones, increasing spillover risk, an effect that is comparable in magnitude, direction, and statistical significance to the effect of fire within the same zones. Results in non-agricultural, non-forest zones remain statistically insignificant. Most notably, in agricultural zones, the spatially lagged ACME becomes negative and nearly significant (*p* = 0.052), suggesting that while fire-driven vegetation loss may not increase local risk, in can raise it in nearby areas through vector spillover. These spatial extensions both reinforce and expend on the core findings and point to important avenues for future research on fire-LULC-zoonosis dynamics.

Future research should investigate potential feedbacks and nonlinearities in fire–LULC–zoonotic disease dynamics. Chronic fire regimes, especially when coupled with drought, soil degradation, or deforestation, may shift ecological thresholds in ways that amplify or reduce zoonotic risk (Santín and Doerr 2016). For example, moderate habitat fragmentation might increase host–vector contact, while extensive fragmentation could lead to biodiversity collapse and lower host availability (Koren and Chaves 2025). Though this study focuses on Africa—where data are available and many deadly zoonoses have emerged—similar LULC patterns in Latin America and Southeast Asia suggest broader relevance (Armenteras et al. 2021; Vadrevu et al. 2019). Cross-regional studies could assess the generalizability of these mechanisms. Finally, because zoonotic risk varies by pathogen—depending on host ecology, transmission mode, and persistence—future models should incorporate host/vector/pathogen-specific dynamics to improve risk prediction and intervention design (Koren et al. 2024).

## Supplementary Information

Below is the link to the electronic supplementary material.

Supplementary file1 (DOCX 1371 KB)

## Data Availability

Raw data and analysis scripts are available as supporting information.
