# Supplementary material for: Fire-Driven Land Cover Change and Zoonotic Disease Risk in African Landscapes
Source: Ecohealth. 2025 Jul 25;22(4):604–12. doi: 10.1007/s10393-025-01743-9 (PMC12628441; doi:10.1007/s10393-025-01743-9)
Supplement: Supplementary file 1 — Supplementary file1 (DOCX 1371 KB) [file 10393_2025_1743_MOESM1_ESM.docx]

Supplementary Information for

**Fire-Driven Land Cover Change and Zoonotic Disease Risk in African Landscapes**

**Additional Data and Methods Information**

**Sample** All data are spatially and temporally harmonized using the AfroGrid framework (Schon & Koren 2022), with the unit of analysis defined as the 0.5-degree grid cell (~55 km × 55 km at the equator) by month, spanning January 2003 to December 2018 (the temporal range over which information on all variables was available). This resolution is widely recommended for analyses using event-based datasets such as G-ZOD (Weidmann 2015) as event-based data often rely on media reports, which may misidentify village names or lack precise coordinates due to access limitations. Aggregating to the 0.5-degree level mitigates these ambiguities, striking a balance between spatial granularity and data reliability. The full panel comprises 10,674 African grid cells observed monthly over the study period, yielding over 2 million observations. Some cell-months are excluded from models with socioeconomic controls due to missing data in sparsely populated areas (e.g., parts of the Sahara Desert). Summary statistics for all variables are provided in Table S1.

**Dependent variable (DV)** The primary measure of zoonotic disease outbreaks is drawn from the Geolocated Zoonotic Outbreak Dataset (GZOD) (Koren & Bukari 2024). To ensure that the dependent variable (DV) serves as a meaningful proxy for zoonotic spillover risk, the analysis includes only outbreaks involving pathogens with strong evidence of animal reservoirs whose habitat and migration patterns are more likely to be affected by fire (Leroy et al. 2005). These include high-impact viruses and bacteria, specifically: Ebola, Marburg, Crimean-Congo Hemorrhagic Fever, bubonic and septicemic plague, rabies, anthrax, and avian and swine influenzas (H1N1, H5N1, H9N1). To reduce the likelihood of false positives, only events confirmed by the World Health Organization, or the International Society for Infectious Diseases were retained, resulting in N=512 outbreak events. For illustration of spatial coverage of the outbreaks, Figure S1 plots an average outbreak frequency map across Africa. The dependent variable is operationalized as the number of confirmed outbreaks involving these pathogens occurring within a given 0.5-degree grid cell during a given month, a resolution aligned with AfroGrid’s spatial framework and selected to mitigate some of the reporting uncertainties outlined above. While this study uses internationally verified outbreak data to improve reliability, uneven surveillance, and underreporting may affect the generalizability of its findings and warrant cautious interpretation. That said, the reliance on internationally verified reports, and the focus on highly virulent and globally monitored pathogens, increases the likelihood of detection even in under-resourced contexts.

**Agricultural areas** Agricultural zones were identified based on the intensively cultivated crops – including cereals (wheat, maize, rice, millet, barley, rye, and sorghum), oil crops (e.g., castor), cotton, sugarcane, sugar beets, bananas, plantains, and cocoa – that exhibit the densest convergence of land use and land cover (LULC), as these are most likely to be reflected in NDVI-based measures (=1, =0 otherwise). These crops represent approximately 45.7% of all grid cell months in the sample. Data on crop production for each grid cell were sourced from the Spatial Production Allocation Model (Yu et al. 2007) for the years 2005, 2010, and 2017 and aggregated into AfroGrid. Monthly values were extrapolated using the last-value-carried-forward approach. A map of the spread of these regions across the continent (at the 0.5-degree resolution) is presented in Figure S1.

**Forest areas** The forest area indicator was developed by identifying 0.5-degree AfroGrid cells with at least 50% forest and jungle coverage (=1, =0 otherwise), using a land classification approach based on Phased Array L-band Synthetic Aperture Radar (PALSAR) images from the Japan Aerospace Exploration Agency (JAXA) (~19.5% of all grid cell months in the sample) (Shimada et al. 2014). JAXA data, with its high precision, is considered state-of-the-art for forest area measurement (Koren & Chaves 2025). The resolution of the PALSAR data is 25m, and images were reprojected to a 0.5-degree resolution using Google Earth Engine to align with the AfroGrid framework. Forest/non-forest coverage information was available for the years 2008–2010 and 2015–2017. For the intervening years, missing data were extrapolated based on the average forest coverage for each grid cell using the last-value-carried-forward approach. Consequently, the forest area indicator is time-constant for each grid cell. The spread of these regions across the continent (at the 0.5-degree resolution) is presented in Figure S1.

**Standardized vegetation (NDVI) anomalies (mediated variable)** To assess the role of land use and land cover (LULC) change in mediating the relationship between fire activity and zoonotic disease outbreaks, I used standardized anomalies in the Normalized Difference Vegetation Index (NDVI) as the primary mediating variable. NDVI data are included in AfroGrid (Schon & Koren 2022) and were sourced using the MODIStsp package (Busetto & Ranghetti 2016). The standardization procedure follows established approaches (Koren & Chaves 2025). For each 0.5-degree grid cell, I calculated the mean and standard deviation of NDVI values across the full study period (January 2003–December 2020). Monthly anomalies were then computed by subtracting each cell’s long-term mean from the observed NDVI and dividing by the cell-specific standard deviation. Unlike unadjusted NDVI, this measure isolates meaningful disturbances relative to local norms, improves comparability across ecosystems (e.g., savanna vs. forest), increases sensitivity to abrupt short-term shocks that are theoretically most relevant for zoonotic spillover processes, and enhances temporal specificity in model estimation. The use of anomalies also aligns with ecological theory emphasizing disruption, rather than static land cover, as a key driver of zoonosis.

**Standardized fire anomalies (mediation treatment)** The mediation treatment was derived from the MODIS Global Fire Product (Justice et al. 2002) and was also included in AfroGrid. Monthly fire detections were aggregated to the 0.5° grid cell level across the study period (Jan. 2003 – Dec. 2018). As done with the NDVI indicator, for each cell, a long-term mean and standard deviation of monthly fire counts were computed. Standardized anomalies were then calculated by subtracting the mean fire count from each monthly value and dividing by the standard deviation. This standardized approach enables direct comparison across diverse ecological zones by capturing relative deviations from typical fire activity, while improving sensitivity to short-term fire shocks. This measure is hence better suited to identifying causal pathways than raw fire counts, which may reflect persistent background conditions rather than disturbances. For comparative purposes, raw NDVI values and fire rate values variation across the continent (averaged over the entire Jan. 2003 – Dec. 2018 period) are presented in Figure S2.

**Nighttime light.** Nighttime light data, used as an indicator of local development and state capacity (Schon & Koren 2022), measures the total number of illuminated pixels within a 0.5-degree grid cell for each year, based on the Defense Meteorological Satellite Program’s Operational Linescan System (DMSP-OLS) system, with corrections applied via the more sensitive (especially in rural areas) Visible Infrared Imaging Radiometer Suite (VIIRS) method (Li et al. 2020). This data is included in the AfroGrid dataset. To account for the wide range of this variable, the data were log-transformed using the log(x+1) approach.

**Population densities.** Population densities were estimated annually for each 0.5-degree grid cell using data from the WorldPop dataset, which provides high-resolution (0.08-degree, or 1 km) population estimates for the years 2000 to 2020, based on a combination of satellite imagery and census data (Tatem 2017). Due to the variable’s range, the data were log-transformed using log(x+1). In combining both “top-down” (satellite) and “bottom-up” (e.g., survey) data sources, the WorldPop dataset is particularly advantageous for analyzing population dynamics in remote and rural areas.

**Placebo Test** To assess whether the observed mediation effects could be attributed to spurious associations rather than land-use and land-cover (LULC) dynamics, placebo tests were conducted using grid cell-months from agricultural, forest, and other zones, restricted to two subsamples: (1) low-population-density cells (<1,000 people) and (2) highly urbanized cells (≥50,000 people). In both contexts, the hypothesized mechanism linking fire-induced vegetation loss to zoonotic spillover should be absent, either due to limited human–vector interaction in remote areas or the physical separation of urban environments from LULC edges (Scasta 2015; Ecke et al. 2019). Mediation models were re-estimated separately for each placebo group using the same specifications described above (See Figure S3 and Table S3 below). These tests serve as a robustness check, helping to isolate the role of LULC-related ecological disturbance from confounding demographic or spatial factors.

**Sensitivity Analyses** To assess the robustness of the mediation findings, we conducted a series of complementary sensitivity tests. First, a targeted analysis was run on a subsample of grid cell-months representing moderately populated areas (1,000–50,000 residents), which were specifically excluded from the placebo conditions sample. These areas are hypothesized to represent zones of heightened zoonotic risk due to their proximity to ecological transitions and sufficient levels of human presence to enable spillover. Second, to address the potential influence of unobserved country-level political or socioeconomic factors, I re-estimated the core models with the inclusion of country-by-year fixed effects. This specification accounts for time-varying national attributes such as governance, economic development, and health system capacity. Third, to evaluate the possibility that country-level spatial clustering – such as shared public health responses or regional environmental shocks – might bias inference, I replicated the main models using country-level clustered standard errors rather than grid-cell clustering. Fourth, to ensure that the results are not driven by environmental confounders related to vegetation changes, I replicated the analysis using controls for Standardized Precipitation Evapotranspiration Index (SPEI), precipitation anomalies, and temperature anomalies in a given cell month using data from CRU TS monthly high-resolution gridded multivariate climate dataset Version 4 (Harris et al. 2020). Across all specifications, the estimated mediation effects remained stable (Table S4-S7 and Figure S4-S7), reinforcing confidence in the localized impacts of fire-driven land-use and land-cover (LULC) change on zoonotic disease risk. I also estimated a geospatial lag model (Table S8 and Figure S8) to test the effects of spatial clustering, although these results are suspect to endogeneity over space and should be interpreted carefully.

**Supplemental Tables and Figures**

**Table S1.** Summary statistics of all relevant variables

| **Variable** | **Min** | **Median** | **Mean** | **Max** | **SD** |
| --- | --- | --- | --- | --- | --- |
| *Zoonotic outbreaks_it_* | 0 | 0 | 0.00023 | 11 | 0.026 |
| *Agricultural areas_it_* | 0 | 0 | 0.457 | 1 | 0.498 |
| *Forest areas_it_* | 0 | 0 | 0.195 | 1 | 0.396 |
| *Vegetation health anomalies_it_* | -7.636 | -0.05 | 0 | 8.82 | 0.998 |
| *Fire anomalies_it_* | -1.022 | -0.009 | 0.09 | 16.387 | 0.793 |
| *Nighttime light_it_ (log)* | 0 | 0 | 1.494 | 5.343 | 1.707 |
| *Population density_it_ (log)* | 0 | 9.746 | 9.051 | 16.733 | 2.98 |
| *SPEI_it_* | -9.004 | -0.186 | -0.274 | 7.887 | 0.990 |
| *Prec. anomalies_it_* | -3.900 | -0.016 | 0.064 | 5.295 | 0.836 |
| *Temp. anomalies_it_* | -4.397 | 0.464 | 0.459 | 5.295 | 0.912 |
| *Zoon. Outbreaks(SPL)_it_* | 0 | 0 | 0.0003 | 2.75 | 0.015 |
| *Veg. health anom.(SPL)_it_* | -4.793 | 0 | -0.0007 | 5.470 | 0.851 |
| *Fire anomalies(SPL)_it_* | -0.657 | -0.080 | 0.139 | 10.890 | 0.715 |


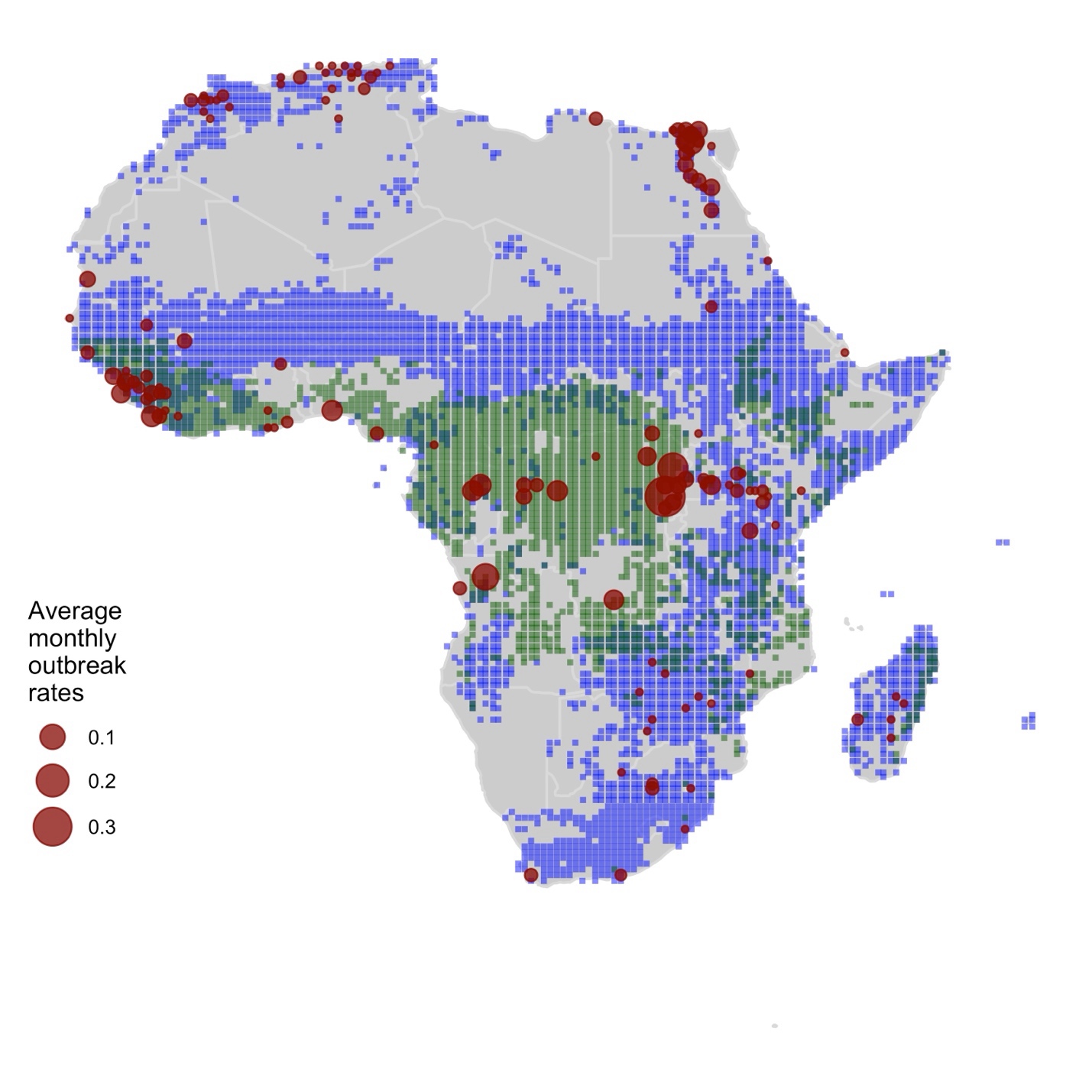


**Figure S1.** Map of zoonotic disease outbreaks (dark red circles), agricultural areas (blue), and forest (green) in Africa by 0.5-degree cells, Jan. 2003 – Dec. 2018.


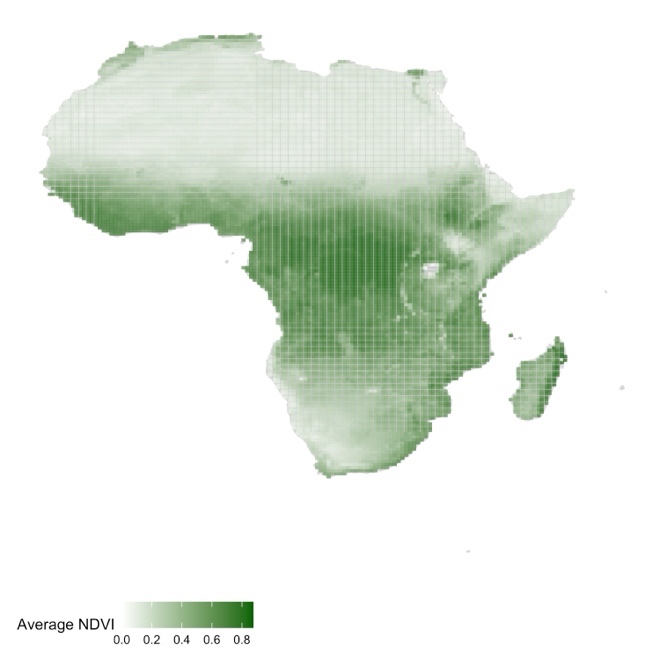

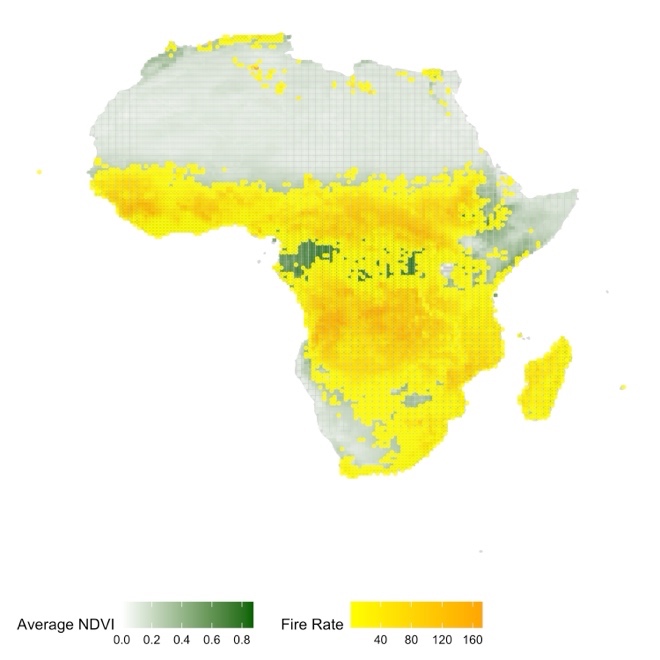


**Figure S2.** Map of average NDVI (top) and average fire rates overlaid over average NDVI (bottom) in Africa by 0.5-degree cells, Jan. 2003 – Dec. 2018.

**Table S2.** Mediation analysis estimates of zoonotic disease outbreaks

|  | **Agricultural** | | **Forest** | | **Other areas** | |
| --- | --- | --- | --- | --- | --- | --- |
| **Variable** | **Eq. (1)** | **Eq. (2)** | **Eq. (1)** | **Eq. (2)** | **Eq. (1)** | **Eq. (2)** |
| *Veg. health anomalies_it_* |  | 0.00002  (0.0003) |  | 0.0001*  (0.00006) |  | 0.00003  (0.00002) |
| *Fire anomalies_it_* | -0.372**  (0.004) | 0.00005  (0.00003) | -0.405**  (0.0001) | 0.0001  (0.0001) | -0.395**  (0.003) | 0.00002  (0.0003) |
| *Zoonotic outbreaks_it-1_* | -0.040  (0.036) | 0.393**  (0.122) | 0.021  (0.046) | 0.444**  (0.046) | -0.0003  (0.031) | 0.372**  (0.070) |
| *Nighttime light_it_ (log)* | 0.0002  (0.001) | 0.0001**  (0.00002) | 0.019**  (0.001) | 0.000003  (0.00003) | 0.007**  (0.001) | 0.0001**  (0.00002) |
| *Population density_it_ (log)* | -0.003**  (0.001) | 0.0002**  (0.00003) | -0.002**  (0.0005) | 0.0001*  (0.00004) | -0.007**  (0.001) | 0.0001**  (0.00003) |
| Constant | -0.014  (0.016) | -0.002**  (0.0004) | -0.452**  (0.022) | -0.001*  (0.0004) | -0.009  (0.012) | -0.002**  (0.0003) |
| N | 556,372 | | 363,494 | | 1,114,130 | |
| R^2^ | 0.131 | 0.149 | 0.298 | 0.198 | 0.158 | 0.137 |
| Adj. R^2^ | 0.131 | 0.149 | 0.298 | 0.197 | 0.158 | 0.137 |

Coefficients are reported with standard errors clustered on grid cell in parentheses. *p<0.05; **p<0.01. Fixed effects by month were included in each regression, though not reported here.

**Table S3.** Placebo test estimates of zoonotic disease outbreaks

|  | **Agricultural** | | **Forest** | | **Other areas** | |
| --- | --- | --- | --- | --- | --- | --- |
| **Variable** | **Eq. (1)** | **Eq. (2)** | **Eq. (1)** | **Eq. (2)** | **Eq. (1)** | **Eq. (2)** |
| *Veg. health anomalies_it_* |  | -0.00001  (0.0001) |  | 0.0001  (0.0001) |  | 0.0001  (0.0001) |
| *Fire anomalies_it_* | -0.406**  (0.008) | 0.0001  (0.0001) | -0.329**  (0.0002) | -0.00003  (0.005) | -0.405**  (0.005) | 0.0001  (0.0001) |
| *Zoonotic outbreaks_it-1_* | -0.041  (0.039) | 0.377**  (0.145) | -0.034  (0.061) | 0.411**  (0.097) | -0.037  (0.039) | 0.355**  (0.090) |
| *Nighttime light_it_ (log)* | -0.007**  (0.002) | 0.0002**  (0.0001) | 0.008**  (0.002) | 0.00005  (0.0001) | -0.002  (0.002) | 0.0002**  (0.0001) |
| *Population density_it_ (log)* | 0.0005  (0.002) | 0.0001**  (0.00003) | -0.0001  (0.001) | 0.0001*  (0.00005) | -0.005**  (0.001) | 0.0001**  (0.00003) |
| Constant | -0.066*  (0.026) | -0.002**  (0.001) | -0.506**  (0.036) | -0.001  (0.0004) | 0.030  (0.018) | -0.002**  (0.0004) |
| N | 178,160 | | 85,554 | | 361,666 | |
| R^2^ | 0.159 | 0.137 | 0.327 | 0.169 | 0.155 | 0.124 |
| Adj. R^2^ | 0.159 | 0.137 | 0.327 | 0.169 | 0.155 | 0.124 |

Coefficients are reported with standard errors clustered on grid cell in parentheses. *p<0.05; **p<0.01. Fixed effects by month were included in each regression, though not reported here.


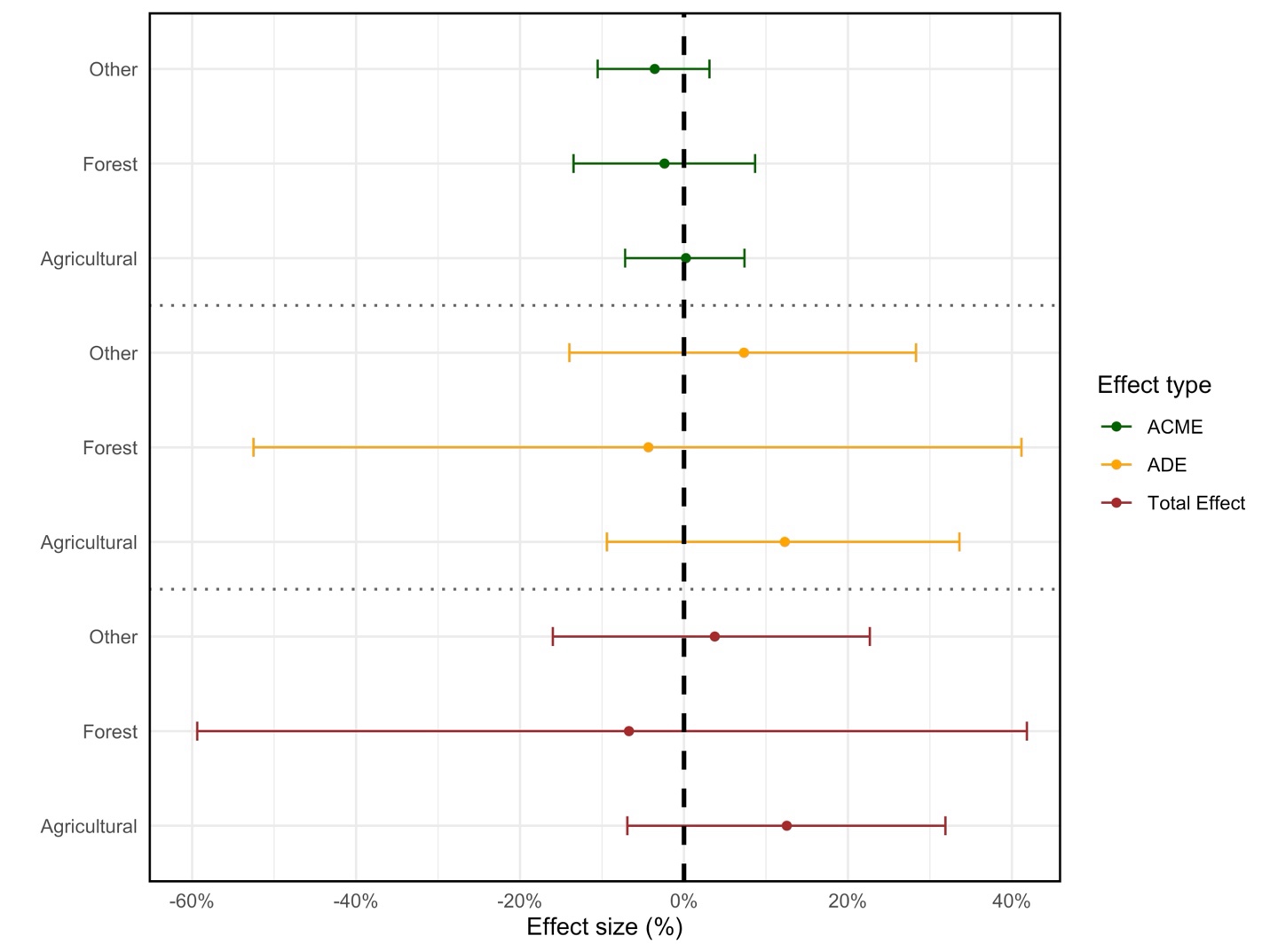


**Figure S3.** Placebo Test Average Causal Mediation Effect as percent change from zoonotic outbreak mean (ACME, top), Average Direct Effect (ADE, center), and Total Effects (bottom) for vegetation anomalies in agricultural, forest, and other (non-agricultural/non-forest) areas in Africa, Jan. 2003 – Dec. 2018. Each figure was estimated based on 1,000 simulations with grid-cell clustered standard errors. N=361,666 (other), N=85,554 (forest), N=178,160 (agricultural). P-values (two-tail tests) are p=0.236 (ACME, other), p=0.678 (ACME, forest), p=0.914 (ACME, agricultural), p=0.520 (ADE, other), p=0.866 (ADE, forest), p=0.262 (ADE, agricultural), p=0.800 (TE, other), p=0.560 (TE, forest), p=0.936 (TE, agricultural).

**Table S4.** High risk case estimates of zoonotic disease outbreaks

|  | **Agricultural** | | **Forest** | | **Other areas** | |
| --- | --- | --- | --- | --- | --- | --- |
| **Variable** | **Eq. (1)** | **Eq. (2)** | **Eq. (1)** | **Eq. (2)** | **Eq. (1)** | **Eq. (2)** |
| *Veg. health anomalies_it_* |  | 0.0003  (0.0003) |  | 0.0002*  (0.0001) |  | 0.00003*  (0.00001) |
| *Fire anomalies_it_* | -0.354**  (0.005) | 0.00003  (0.00002) | -0.425**  (0.007) | 0.0001*  (0.0001) | -0.390**  (0.004) | 0.00002  (0.00002) |
| *Zoonotic outbreaks_it-1_* | -0.043  (0.099) | 0.500**  (0.067) | 0.084  (0.057) | 0.482  (0.023) | 0.158**  (0.045) | 0.438**  (0.028) |
| *Nighttime light_it_ (log)* | 0.003*  (0.001) | -0.000003  (0.00001) | 0.023**  (0.001) | -0.00002  (0.00002) | 0.010**  (0.001) | -0.00001  (0.0001) |
| *Population density_it_ (log)* | -0.004*  (0.002) | -0.00001  (0.00002) | 0.005**  (0.001) | 0.00004  (0.0001) | -0.003**  (0.001) | 0.00002  (0,00002) |
| Constant | 0.006  (0.022) | 0.0001  (0.0002) | -0.524**  (0.028) | -0.0003  (0.001) | -0.067**  (0.016) | -0.0002  (0.0002) |
| N | 373,212 | | 277,490 | | 752,464 | |
| R^2^ | 0.121 | 0.250 | 0.299 | 0.233 | 0.161 | 0.192 |
| Adj. R^2^ | 0.121 | 0.250 | 0.299 | 0.233 | 0.161 | 0.192 |

Coefficients are reported with standard errors clustered on grid cell in parentheses. *p<0.05; **p<0.01. Fixed effects by month were included in each regression, though not reported here.


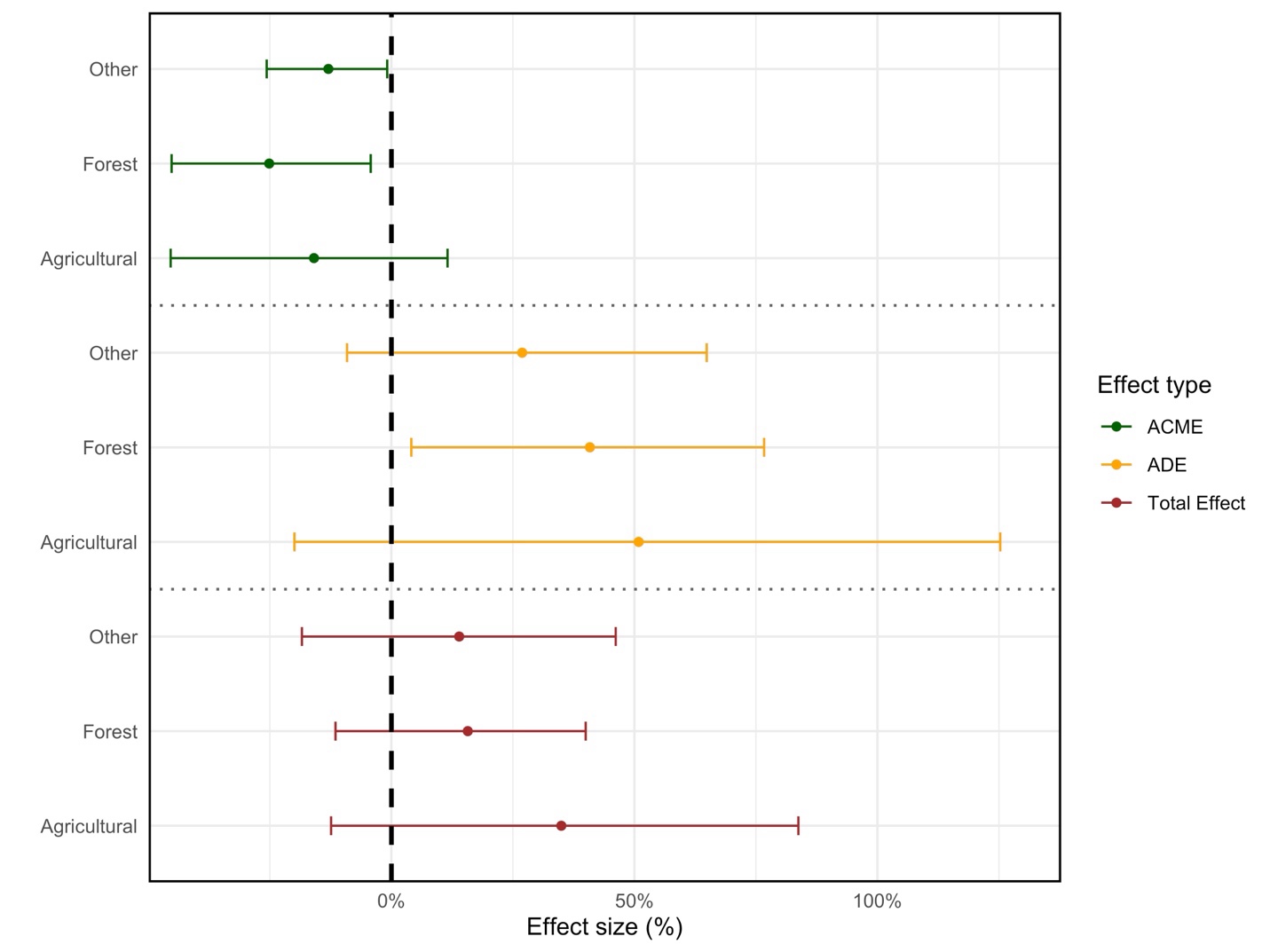


**Figure S4.** High risk sample Average Causal Mediation Effect as percent change from zoonotic outbreak mean (ACME, top), Average Direct Effect (ADE, center), and Total Effects (bottom) for vegetation anomalies in agricultural, forest, and other (non-agricultural/non-forest) areas in Africa, Jan. 2003 – Dec. 2018. Each figure was estimated based on 1,000 simulations with grid-cell clustered standard errors. N=752,464 (other), N=277,490 (forest), N=373,212 (agricultural). P-values (two-tail tests) are p=0.044 (ACME, other), p=0.016 (ACME, forest), p=0.268 (ACME, agricultural), p=0.160 (ADE, other), p=0.030 (ADE, forest), p=0.172 (ADE, agricultural), p=0.428 (TE, other), p=0.256 (TE, forest), p=0.192 (TE, agricultural).

**Table S5.** Country and year confounder adjusted estimates of zoonotic disease outbreaks

|  | **Agricultural** | | **Forest** | | **Other areas** | |
| --- | --- | --- | --- | --- | --- | --- |
| **Variable** | **Eq. (1)** | **Eq. (2)** | **Eq. (1)** | **Eq. (2)** | **Eq. (1)** | **Eq. (2)** |
| *Veg. health anomalies_it_* |  | 0.00002  (0.00003) |  | 0.0001*  (0.00006) |  | 0.00004  (0.00002) |
| *Fire anomalies_it_* | -0.374**  (0.004) | 0.00005  (0.00003) | -0.404**  (0.007) | 0.0001  (0.0001) | -0.396**  (0.003) | 0.00003  (0.00003) |
| *Zoonotic outbreaks_it-1_* | -0.039  (0.036) | 0.392**  (0.122) | 0.023  (0.037) | 0.444**  (0.046) | -0.006  (0.032) | 0.372**  (0.070) |
| *Nighttime light_it_ (log)* | 0.005**  (0.001) | -0.000004  (0.00002) | 0.006**  (0.001) | 0.00003  (0.0001) | 0.008**  (0.001) | -0.000001  (0.00002) |
| *Population density_it_ (log)* | -0.003  (0.001) | 0.0002**  (0.00005) | 0.002**  (0.0005) | 0.0001*  (0.0001) | -0.006**  (0.001) | 0.0002**  (0.0001) |
| Constant | -0.036*  (0.017) | -0.002**  (0.001) | -0.472**  (0.027) | -0,001  (0.001) | -0.081**  (0.012) | -0.003**  (0.001) |
| N | 553,877 | | 363,302 | | 1,109,223 | |
| R^2^ | 0.134 | 0.150 | 0.300 | 0.198 | 0.162 | 0.137 |
| Adj. R^2^ | 0.134 | 0.150 | 0.300 | 0.198 | 0.162 | 0.137 |

Coefficients are reported with standard errors clustered on grid cell in parentheses. *p<0.05; **p<0.01. Fixed effects by month, country, and year were included in each regression, though not reported here.


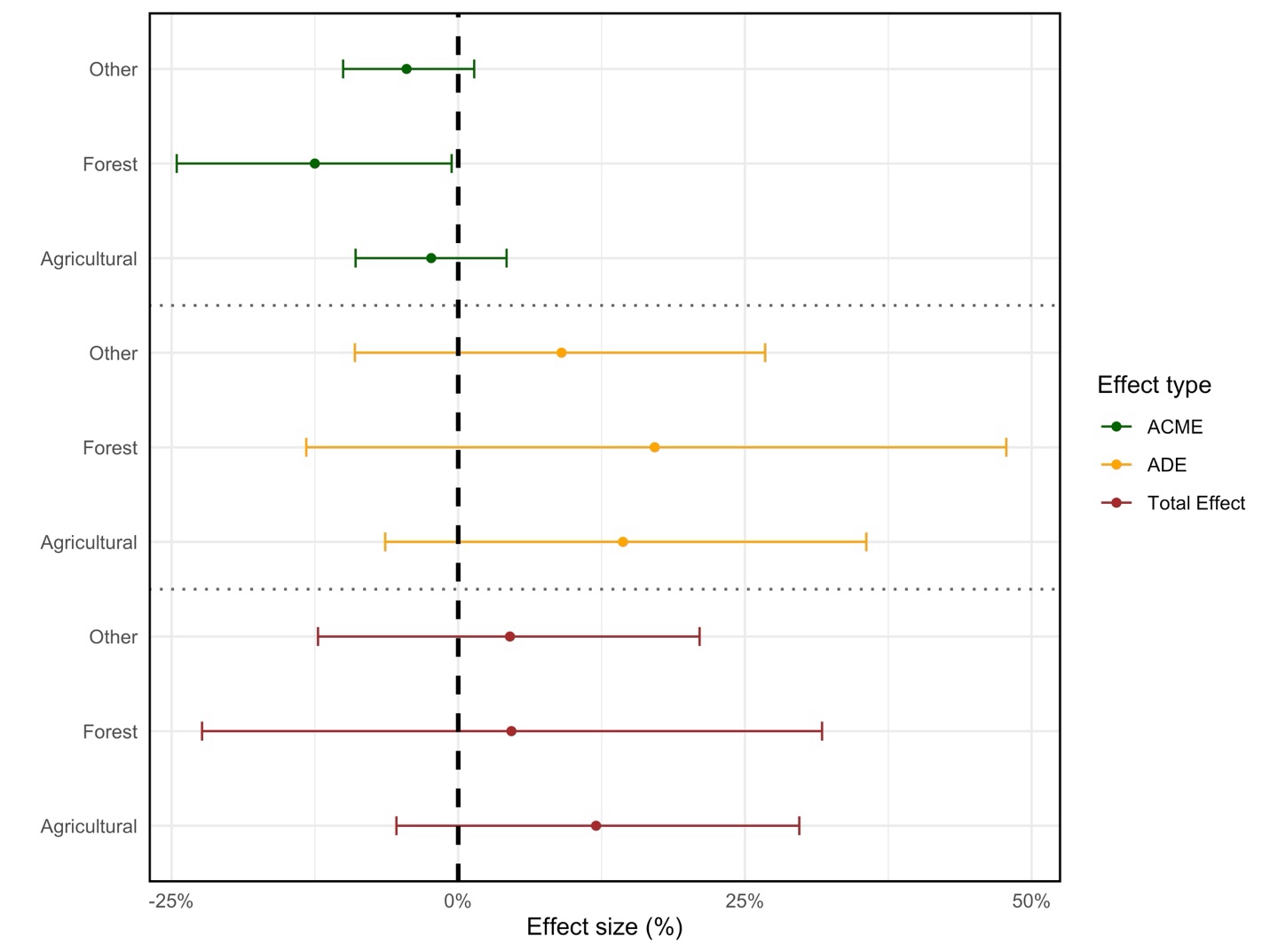


**Figure S5.** Country and year adjusted Average Causal Mediation Effect as percent change from zoonotic outbreak mean (ACME, top), Average Direct Effect (ADE, center), and Total Effects (bottom) for vegetation anomalies in agricultural, forest, and other (non-agricultural/non-forest) areas in Africa, Jan. 2003 – Dec. 2018. Each figure was estimated based on 1,000 simulations with grid-cell clustered standard errors. N=1,109,223 (other), N=363,302 (forest), N=553,877 (agricultural). P-values (two-tail tests) are p=0.152 (ACME, other), p=0.042 (ACME, forest), p=0.564 (ACME, agricultural), p=0.300 (ADE, other), p=0.262 (ADE, forest), p=0.160 (ADE, agricultural), p=0.656 (TE, other), p=0.762 (TE, forest), p=0.546 (TE, agricultural).

**Table S6.** Country heterogeneity adjusted estimates of zoonotic disease outbreaks

|  | **Agricultural** | | **Forest** | | **Other areas** | |
| --- | --- | --- | --- | --- | --- | --- |
| **Variable** | **Eq. (1)** | **Eq. (2)** | **Eq. (1)** | **Eq. (2)** | **Eq. (1)** | **Eq. (2)** |
| *Veg. health anomalies_it_* |  | 0.00002  (0.00003) |  | 0.0001*  (0.00006) |  | 0.00004  (0.00003) |
| *Fire anomalies_it_* | -0.372**  (0.030) | 0.00005  (0.00004) | -0.404**  (0.043) | 0.0001  (0.0001) | -0.395**  (0.026) | 0.00003  (0.00003) |
| *Zoonotic outbreaks_it-1_* | -0.040  (0.043) | 0.393**  (0.145) | 0.021  (0.048) | 0.444**  (0.053) | -0.002  (0.049) | 0.372**  (0.096) |
| *Nighttime light_it_ (log)* | 0.0002  (0.003) | 0.0001  (0.00004) | 0.019**  (0.005) | 0.000003  (0.00003) | 0.007  (0.004) | 0.0001  (0.00004) |
| *Population density_it_ (log)* | -0.003  (0.003) | 0.0002*  (0.0001) | -0.002  (0.002) | 0.0001  (0.0001) | -0.007  (0.003) | 0.0001**  (0.00004) |
| Constant | -0.014  (0.106) | -0.002*  (0.001) | -0.453*  (0.195) | -0.001  (0.001) | -0.010  (0.112) | -0.002**  (0.001) |
| N | 553,877 | | 363,302 | | 1,109,223 | |
| R^2^ | 0.131 | 0.149 | 0.298 | 0.198 | 0.158 | 0.137 |
| Adj. R^2^ | 0.131 | 0.149 | 0.298 | 0.198 | 0.158 | 0.137 |

Coefficients are reported with standard errors clustered on country in parentheses. *p<0.05; **p<0.01. Fixed effects by month were included in each regression, though not reported here.


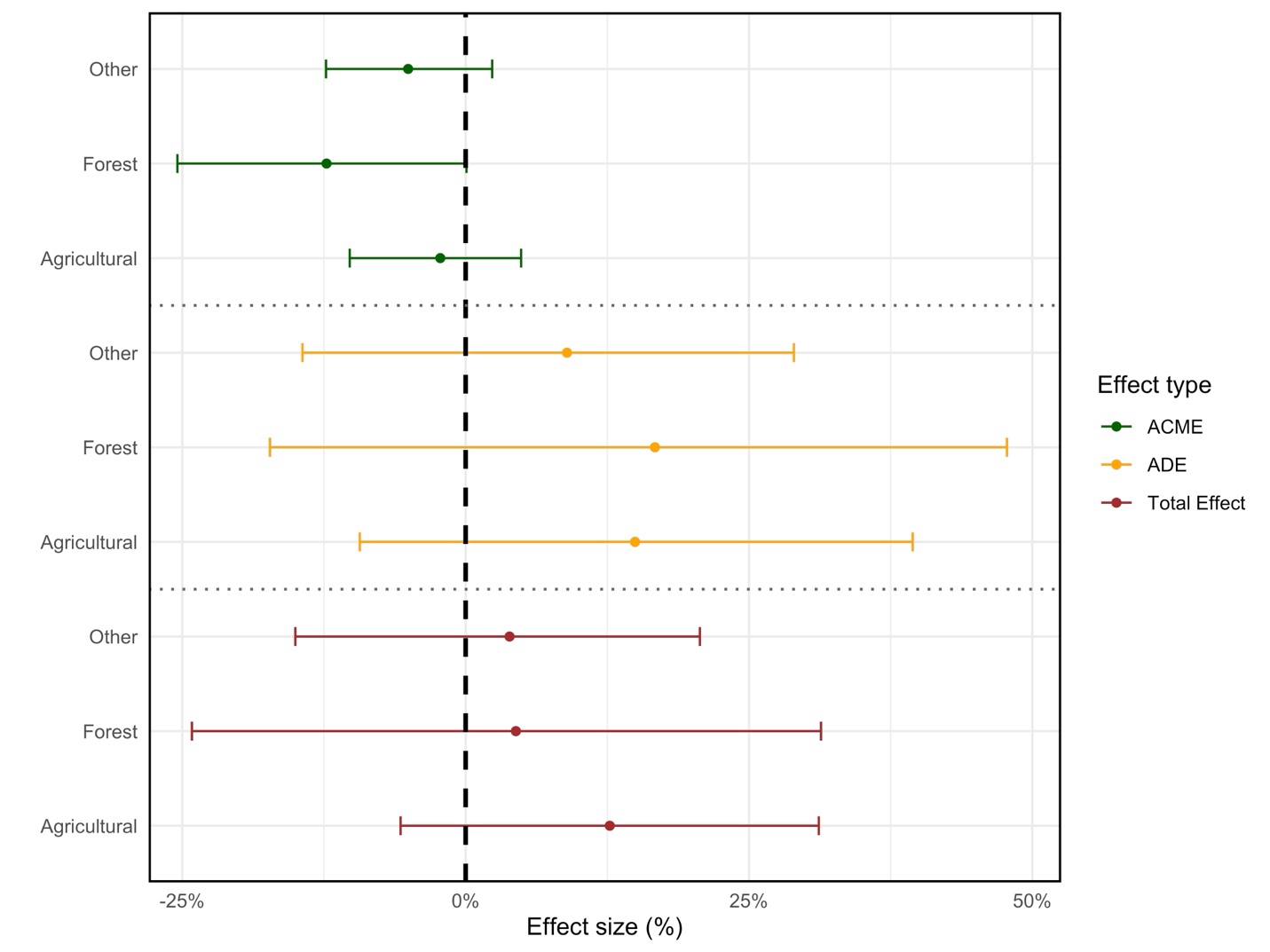


**Figure S6.** Country-heterogeneity adjusted Average Causal Mediation Effect as percent change from zoonotic outbreak mean (ACME, top), Average Direct Effect (ADE, center), and Total Effects (bottom) for vegetation anomalies in agricultural, forest, and other (non-agricultural/non-forest) areas in Africa, Jan. 2003 – Dec. 2018. Each figure was estimated based on 1,000 simulations with country clustered standard errors. N=1,109,223 (other), N=363,302 (forest), N=553,877 (agricultural). P-values (two-tail tests) are p=0.204 (ACME, other), p=0.016 (ACME, forest), p=0.584 (ACME, agricultural), p=0.432 (ADE, other), p=0.314 (ADE, forest), p=0.204 (ADE, agricultural), p=0.580 (TE, other), p=0.740 (TE, forest), p=0.504 (TE, agricultural).

**Table S7.** Environmentally adjusted estimates of zoonotic disease outbreaks

|  | **Agricultural** | | **Forest** | | **Other areas** | |
| --- | --- | --- | --- | --- | --- | --- |
| **Variable** | **Eq. (1)** | **Eq. (2)** | **Eq. (1)** | **Eq. (2)** | **Eq. (1)** | **Eq. (2)** |
| *Veg. health anomalies_it_* |  | 0.00002  (0.0004) |  | 0.0001*  (0.00006) |  | 0.00004  (0.00003) |
| *Fire anomalies_it_* | -0.386**  (0.005) | 0.0001  (0.00004) | -0.404**  (0.007) | 0.0001  (0.0001) | -0.410**  (0.004) | 0.00005  (0.0003) |
| *Zoonotic outbreaks_it-1_* | -0.026  (0.036) | 0.428**  (0.116) | 0.019  (0.038) | 0.444**  (0.046) | 0.008  (0.031) | 0.395**  (0.068) |
| *Nighttime light_it_ (log)* | -0.009**  (0.001) | 0.0001**  (0.00002) | 0.019**  (0.001) | 0.00001  (0.00003) | 0.002**  (0.001) | 0.0001**  (0.00002) |
| *Population density_it_ (log)* | 0.001  (0.001) | 0.0002**  (0.00004) | -0.003**  (0.001) | 0.0001*  (0.00004) | -0.002**  (0.001) | 0.0001**  (0.00003) |
| *SPEI_it_* | 0.093**  (0.005) | 0.0001*  (0.0001) | 0.028**  (0.006) | -0.0002  (0.0001) | 0.085**  (0.004) | 0.0001  (0.0001) |
| *Prec. anom_it_* | -0.012**  (0.004) | -0.0002*  (0.0001) | 0.007  (0.007) | 0.0002*  (0.0001) | -0.021**  (0.003) | 0.0001  (0.0001) |
| *Temp. anom_it_* | -0.111**  (0.002) | 0.0001  (0.0001) | -0.049**  (0.002) | -0.00004  (0.0001) | -0.091**  (0.002) | 0.00005  (0.00004) |
| Constant | 0.190**  (0.019) | -0.002**  (0.0004) | -0.424**  (0.023) | -0.001*  (0.0004) | 0.088**  (0.013) | -0.002**  (0.0003) |
| N | 478,468 | | 394,052 | | 987,376 | |
| R^2^ | 0.155 | 0.174 | 0.289 | 0.198 | 0.171 | 0.150 |
| Adj. R^2^ | 0.155 | 0.174 | 0.289 | 0.197 | 0.171 | 0.150 |

Coefficients are reported with standard errors clustered on grid cell in parentheses. *p<0.05; **p<0.01. Fixed effects by month were included in each regression, though not reported here.


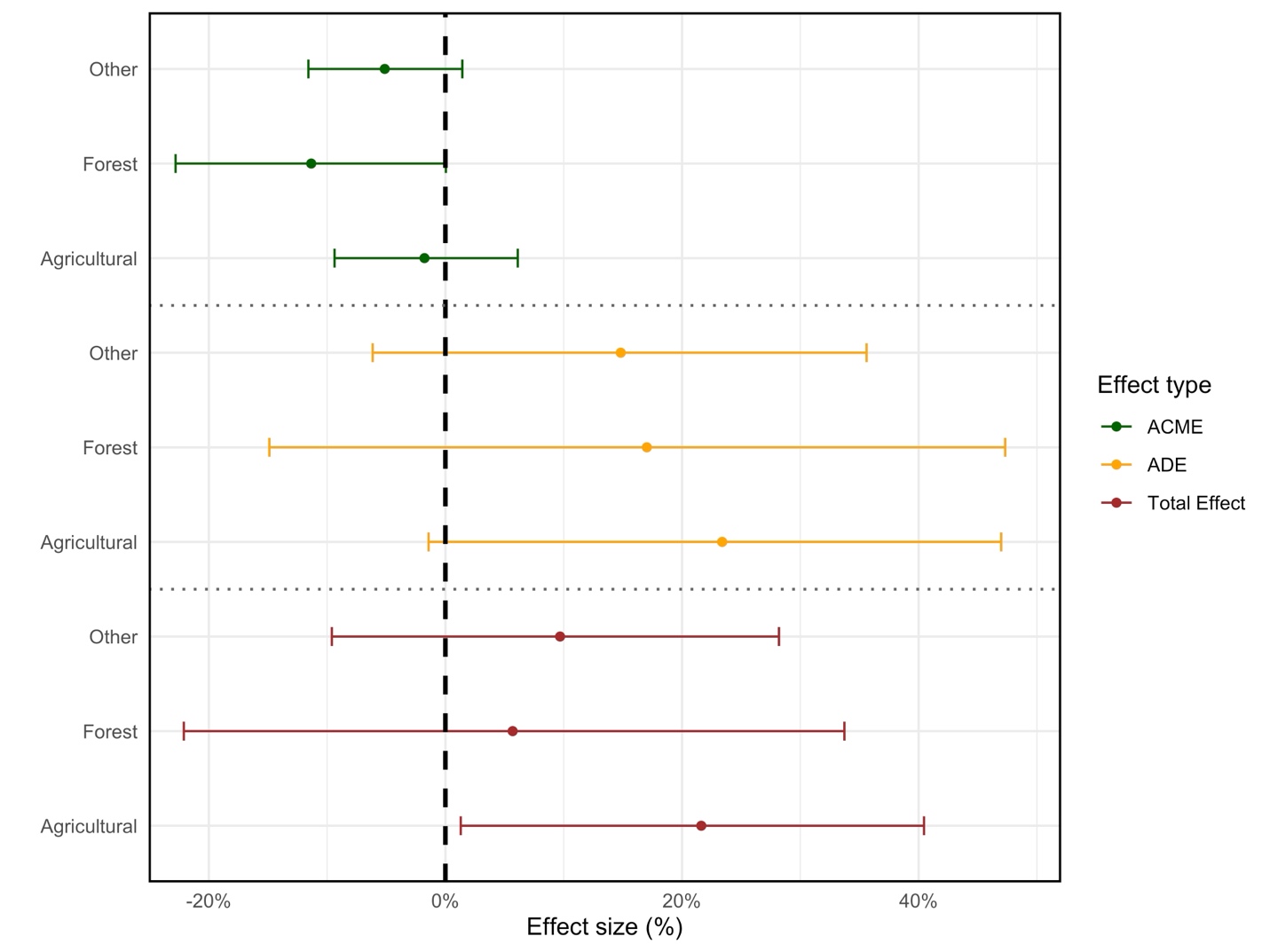


**Figure S7.** Environmentally adjusted Average Causal Mediation Effect as percent change from zoonotic outbreak mean (ACME, top), Average Direct Effect (ADE, center), and Total Effects (bottom) for vegetation anomalies in agricultural, forest, and other (non-agricultural/non-forest) areas in Africa, Jan. 2003 – Dec. 2018. Each figure was estimated based on 1,000 simulations with grid-cell clustered standard errors. N=987,376 (other), N=394,052 (forest), N=478,468 (agricultural). P-values (two-tail tests) are p=0.120 (ACME, other), p=0.052 (ACME, forest), p=0.674 (ACME, agricultural), p=0.136 (ADE, other), p=0.290 (ADE, forest), p=0.070 (ADE, agricultural), p=0.340 (TE, other), p=0.740 (TE, forest), p=0.340 (TE, agricultural).

**Table S8.** Spatial lag model estimates of zoonotic disease outbreaks

|  | **Agricultural** | | **Forest** | | **Other areas** | |
| --- | --- | --- | --- | --- | --- | --- |
| **Variable** | **Eq. (1)** | **Eq. (2)** | **Eq. (1)** | **Eq. (2)** | **Eq. (1)** | **Eq. (2)** |
| *Veg. health anom.(SPL)_it_* |  | 8.8e-05*  (4.4e-05) |  | 0.0001*  (0.00006) |  | 0.00003  (0.00005) |
| *Fire anomalies(SPL)_it_* | -0.647**  (0.005) | 0.00014*  (0.00006) | -0.590**  (0.007) | 0.00004  (0.00005) | -0.632**  (0.003) | -3.7e-06 (0.00005) |
| *Zoonotic outbreaks_it-1_* | 0.061  (0.033) | 0.392**  (0.122) | 0.047  (0.028) | 0.444**  (0.046) | 0.039  (0.023) | 0.372**  (0.070) |
| *Zoon. Outbreaks(SPL)_it_* | 0.056  (0.066) | 0.049*  (0.020) | 0.123  (0.074) | 0.019*  (0.092) | 0.173**  (0.050) | 0.026**  (0.009) |
| *Nighttime light_it_ (log)* | -0.003**  (0.0008) | 0.00007**  (0.00002) | 0.011**  (0.0007) | 2.5e-06 (0.00003) | 0.0002  (0.0004) | 0.000006**  (0.00002) |
| *Population density_it_ (log)* | -0.0002  (0.001) | 0.0002**  (0.00004) | -0.002**  (0.0004) | 0.0001*  (0.00004) | 0.0006  (0.0004) | 0.00014**  (0.00003) |
| Constant | -0.014  (0.013) | -0.002**  (0.0004) | -0.239**  (0.019) | -0,001*  (0.0004) | -0.014  (0.008) | -0.002**  (0.0003) |
| N | 518,380 | | 345,998 | | 1,041,482 | |
| R^2^ | 0.255 | 0.150 | 0.427 | 0.198 | 0.198 | 0.288 |
| Adj. R^2^ | 0.255 | 0.150 | 0.427 | 0.198 | 0.198 | 0.288 |

Coefficients are reported with standard errors clustered on grid cell in parentheses. *p<0.05; **p<0.01. Fixed effects by month were included in each regression, though not reported here.


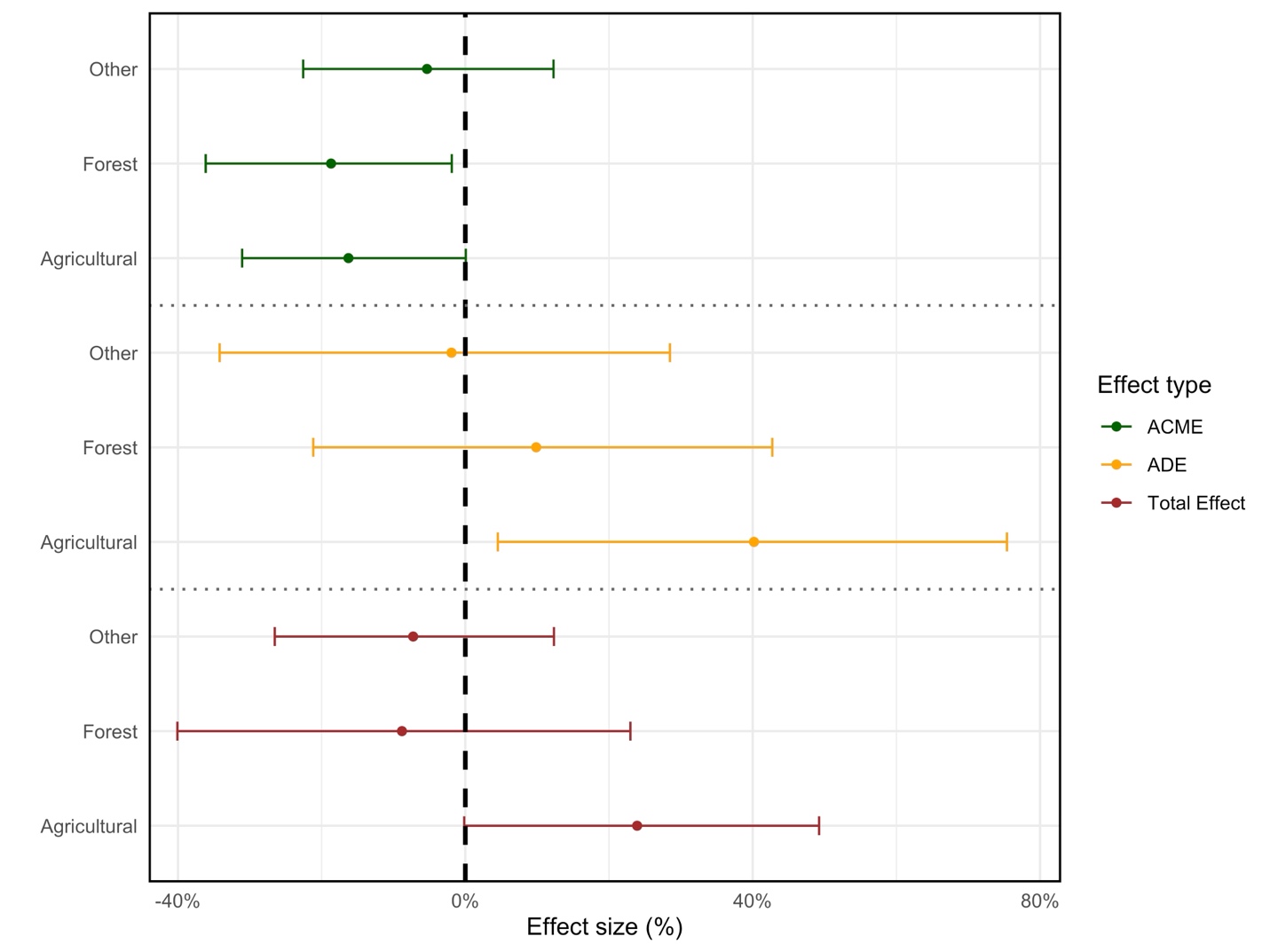


**Figure S8.** Spatially Lagged Average Causal Mediation Effect as percent change from zoonotic outbreak mean (ACME, top), Average Direct Effect (ADE, center), and Total Effects (bottom) for vegetation anomalies in agricultural, forest, and other (non-agricultural/non-forest) areas in Africa, Jan. 2003 – Dec. 2018. Each figure was estimated based on 1,000 simulations with grid-cell clustered standard errors. N=1,041,482 (other), N=345,998 (forest), N=518,380 (agricultural). P-values (two-tail tests) are p=0.568 (ACME, other), p=0.028 (ACME, forest), p=0.052 (ACME, agricultural), p=0.880 (ADE, other), p=0.592 (ADE, forest), p=0.030 (ADE, agricultural), p=0.828 (TE, other), p=0.588 (TE, forest), p=0.076 (TE, agricultural).

**References**

Busetto, L. & Ranghetti, L. MODIStsp: An R package for automatic preprocessing of MODIS Land Products time series. Comput. Geosci. 97, 40–48 (2016).

Ecke, F. et al. Wildfire-induced short-term changes in a small mammal community increase prevalence of a zoonotic pathogen? Ecol. Evol. 9, 12459–12470 (2019).

Harris, I., Osborn, T. J., Jones, P. & Lister, D. Version 4 of the CRU TS monthly high resolution gridded multivariate climate dataset. Sci. Data 7, 1–18 (2020).

Koren, O. & Bukari, K. N. (Re) Emerging disease and conflict risk in Africa, 1997–2019. Nat. Hum. Behav. 8, 1506–1513 (2024).

Koren, O., & Chaves, L. F. The land-use land-cover change–emerging infectious disease nexus reconsidered. *BioScience*, biaf045 (2025).

Li, X., Zhou, Y., Zhao, M. & Zhao, X. A harmonized global nighttime light dataset 1992–2018. Sci. Data 7, 168 (2020).

Scasta, J. D. Fire and parasites: an under-recognized form of anthropogenic land use change and mechanism of disease exposure. EcoHealth 12, 398–403 (2015).

Schon, J. & Koren, O. Introducing AfroGrid, a unified framework for environmental conflict research in Africa. Sci. Data 9, 116 (2022).

Shimada, M. et al. New Global Forest/Non-forest Maps from ALOS PALSAR Data (2007–2010). Remote Sens. Environ. 155, 13–31 (2014).

Tatem, A. J. Worldpop, open data for spatial demography. Sci. Data 4, 1–4 (2017).

Weidmann, N. B. On the accuracy of media-based conflict event data. J. Conflict Resolut. 59, 1129–1149 (2015).

Yu, Q. et al. A cultivated planet in 2010–Part 2: the global gridded agricultural-production maps. Earth Syst. Sci. Data 12, 3545–3572 (2020).
